# Supplementary material for: MetaRibo-Seq measures translation in microbiomes
Source: Nat Commun. 2020 Jun 29;11:3268. doi: 10.1038/s41467-020-17081-z (PMC7324362; doi:10.1038/s41467-020-17081-z)
Supplement: Supplementary file 10 — Supplementary Data 7 [file 41467_2020_17081_MOESM10_ESM.zip › File2/Confidence_High_Taxonomy/179185_out.krona.html]

Javascript must be enabled to view this page.

members
magnitude
magnitudeUnassigned
count
unassigned
taxon
rank

179185\_out

5

5
superkingdom
2

5
976
phylum

4
class
117743

order
200644
4

4
49546
family

1016
genus
4

1
1017

SRS147281\_contig\_number\_18362
species

species

SRS012989\_contig\_number\_3676SRS015899\_contig\_number\_10917SRS022083\_contig\_number\_11156
1019
3


SRS049268\_contig\_number\_29713
652708
species
1
